# Peutz–Jeghers syndrome with polyps in the stomach, duodenum, and small and large intestine: a case report

**DOI:** 10.1186/s13256-023-04335-9

**Published:** 2024-03-05

**Authors:** Zaryab Ali Shah, Muhammad Zeb, Muhammad Ilyas, Hasnain Hamid, Komal Fatima, Maria Batool, Muhammad Abbas

**Affiliations:** 1 Hayatabad Medical Complex Peshawar, Peshawar, Pakistan; 2 General Surgery, Hayatabad Medical Complex, Peshawar, Pakistan

**Keywords:** Peutz–Jeghers, Hamartomatous, Polyp

## Abstract

**Background:**

Peutz–Jeghers syndrome is a rare hereditary condition characterized by gastrointestinal polyps and pigmented oral lesions. The case contributes to a deeper understanding of Peutz–Jeghers syndrome and underscores the significance of interdisciplinary collaboration for accurate diagnosis and tailored therapeutic strategies.

**Case description:**

We present a case of a 15-year-old Afghan female patient with multiple polyps throughout the gastrointestinal tract and mucocutaneous pigmentation. Despite previous medical visits and colonoscopies, her symptoms persisted. A multidisciplinary team discussed the case and recommended further investigations and interventions. A polypectomy was performed, confirming the presence of hamartomatous polyps. The patient was diagnosed with Peutz–Jeghers syndrome, but during the course of treatment she went through complications and was managed surgically as well.

**Conclusion:**

Timely polyp removal and lifelong surveillance are crucial in managing Peutz–Jeghers syndrome. Further research and genetic analysis are needed to improve understanding and management of this rare disorder.

## Introduction

Peutz–Jeghers syndrome is a rare hereditary autosomal condition that causes gastrointestinal hamartomatous polyps and buccal pigment lesions [1]. Mucocutaneous pigmentations appears in 95% of individuals with the disorder [2]. Polyps can be as little as a few microns or as large as 7 cm [3]. According to estimates, it affects 1 in 100,000 people [4].

A germline mutation in the serine/threonine kinase 11 or liver kinase B1 (*STK11/LKB1*) genes causes it to affect both men and women [4, 5]. Men and women are typically diagnosed at ages 23 years and 26 years, respectively [2, 4].

The median age at which a symptom manifests is 13 years, indicating that they frequently manifest at a young age. More than 50% of people will experience symptoms by the age of 20 years [6].

Preoperative diagnosis is frequently missed or delayed due to non-specific symptoms in the absence of a pathognomonic clinical picture [7].

Bleeding, bowel obstruction, and intussusception are common complications in patients with PJS [8].

Patients with PJS are predisposed to a variety of complications and malignancies, particularly gastrointestinal and breast cancers.^22^ As a result, patients must be monitored regularly to avoid complications and improve their outcomes. The removal of larger polyps and periodic surveillance are intended to reduce the occurrence of complications in PJS [9].

Here we present a case of young female patient with multiple hamartomatous polyps involving the duodenum, small intestine, and large intestine with mucocutaneous pigmentation on lips and below the lower eyelids, which was managed through a multidisciplinary approach.

## Case description

This work has been reported in line with the SCARE criteria [7].

A Afghan female patient aged 15 years was admitted to the medical ward with bleeding per rectum (melena) during defecation, which had been occurring for the past 6 months. She also had generalized body ache for the past 3 years. Additionally, she had black pigmentations on the inner side of her lower lip and right lower eyelid since childhood. Over the past 3 years, she had also experienced intermittent left flank pain. However, her symptoms had shown no improvement thus far. The patient reported having multiple visits to medical and gastroenterology specialists over the past 2 years. She was initially admitted for complaints of left flank pain and her first colonoscopy 1.5 years ago. Subsequently, three more colonoscopies were performed until now, and on all the colonoscopies, provisional diagnosis of PJS was made. The patient has a positive family history of perioral pigmentation (her father, brother, and niece). Her father underwent exploratory laparotomy for an undisclosed gut pathology, but unfortunately, did not survive the procedure. Additionally, her brother underwent subtotal colectomy 6 months ago for similar condition.

**Fig. 1:**
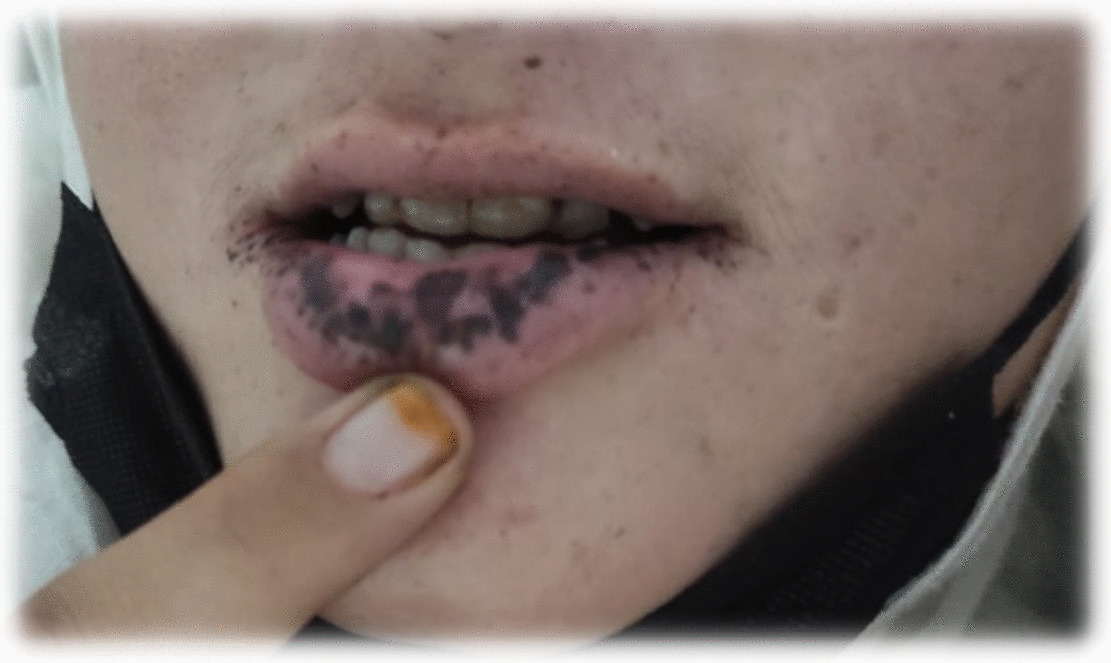
Mucocutaneous pigmentation on lower lip

Upon general physical examination, the patient appeared pale and vitally stable, and the only notable finding was the black pigmentation on the inner side of her lower lip (refer to Fig. 1) and her right lower eyelids. Per rectal examination was unremarkable. The systemic examination did not reveal any abnormalities.

Baseline investigations showed that her hemoglobin (Hb) was 10.5 g/dl (normal above 12.5 g/dl). (Please note that she had multiple histories of blood transfusion and the most recent one was 3 weeks previously).

Ultrasound was also normal with no positive findings.

Computed tomography (CT) scan showed multiple jejunal polypoid lesion forming at least two intussusception, antral, pyloric, and descending colon polyps.

Her previous colonoscopy results are all summarized in Table 1.

**Table 1:** Colonoscopy findings

Date	Colonoscopy findings
13/9/2022	One giant polyp in mid transverse colon, and multiple small ones from rectum to mid transverse colon. Scope nonnegotiable past mid transverse
26/9/2022	Cecum normal. Ascending colon normal. Multiple small polyps of variable size noted from rectum to hepatic flexure. One giant polyp in mid transverse colon removed with suture
1/11/2022	Giant polyp in proximal transverse colon causing intussusceptions of colon, which was relieved endoscopically. After that, up to hepatic flexure investigated. Giant polyp not amenable to resection due to broad and large stalk. Multiple small polyps in descending and sigmoid colon. Few small polypectomies done
9/6/2023	One large polyp in the cecum. One in transverse colon. Another last in the splenic flexure occupying whole lumen. One in the descending colon. Three small ones in the rectum and sigmoid. No polypectomy done this time

On the basis of the above findings, the patient was diagnosed as provisional case of PJS and was discussed in multidisciplinary meeting (consisting of physician, general surgeon, gastroenterologist, radiologist, and oncologist). Multidisciplinary team (MDT) decided to do redo colonoscopy and endoscopy for histopathology and CT enterography for further assessment of bowel.

**Fig. 2:**
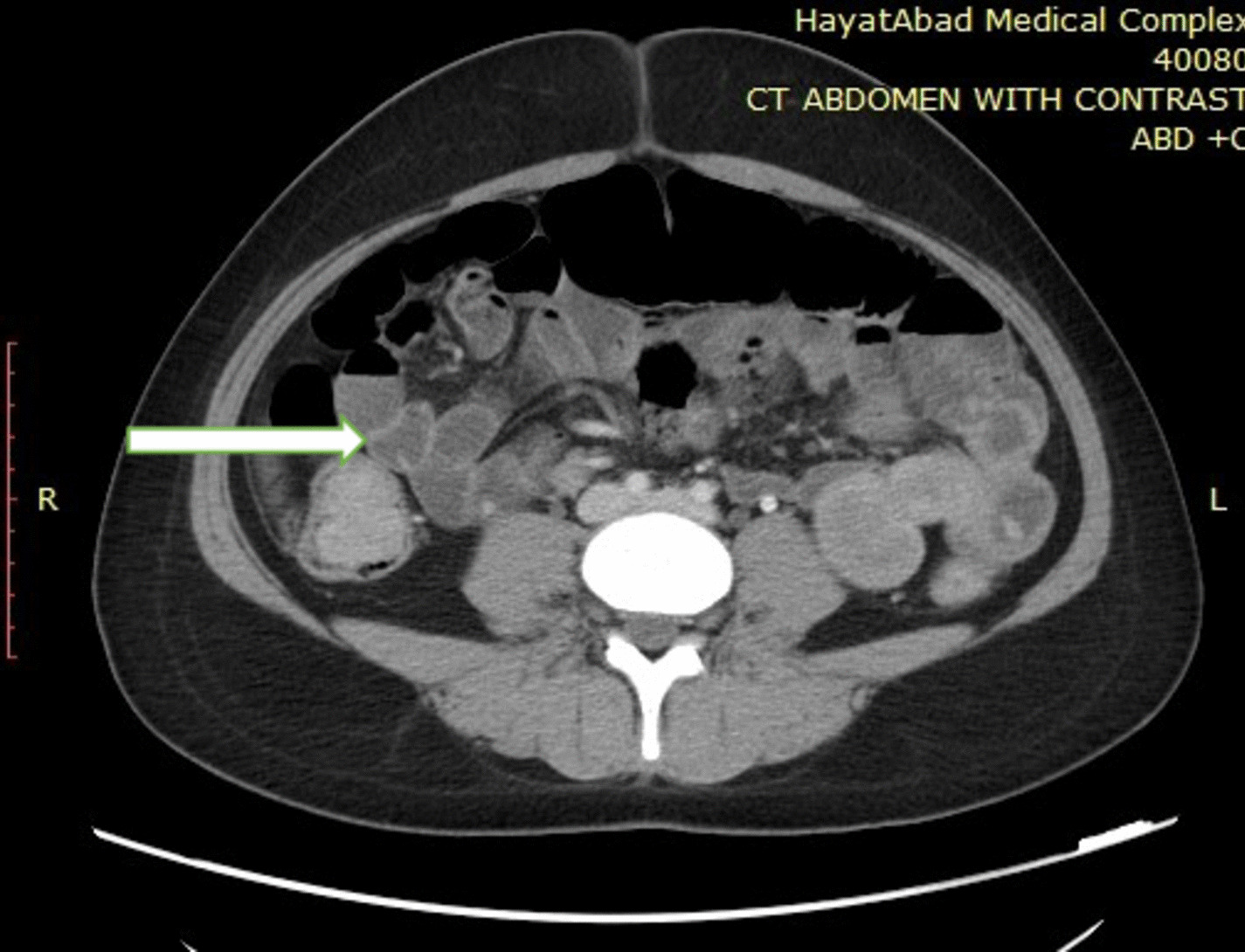
Computed tomography scan with contrast of abdomen and pelvis showing intussusception in small bowel and polyps (arrow)

On CT enterography, multiple polyps involving the pylorus, duodenum, jejunum, and large bowel were found with no signs of obstruction. One polyp in the jejunum was causing short segment intussusception (Fig. 2).

**Fig. 3:**
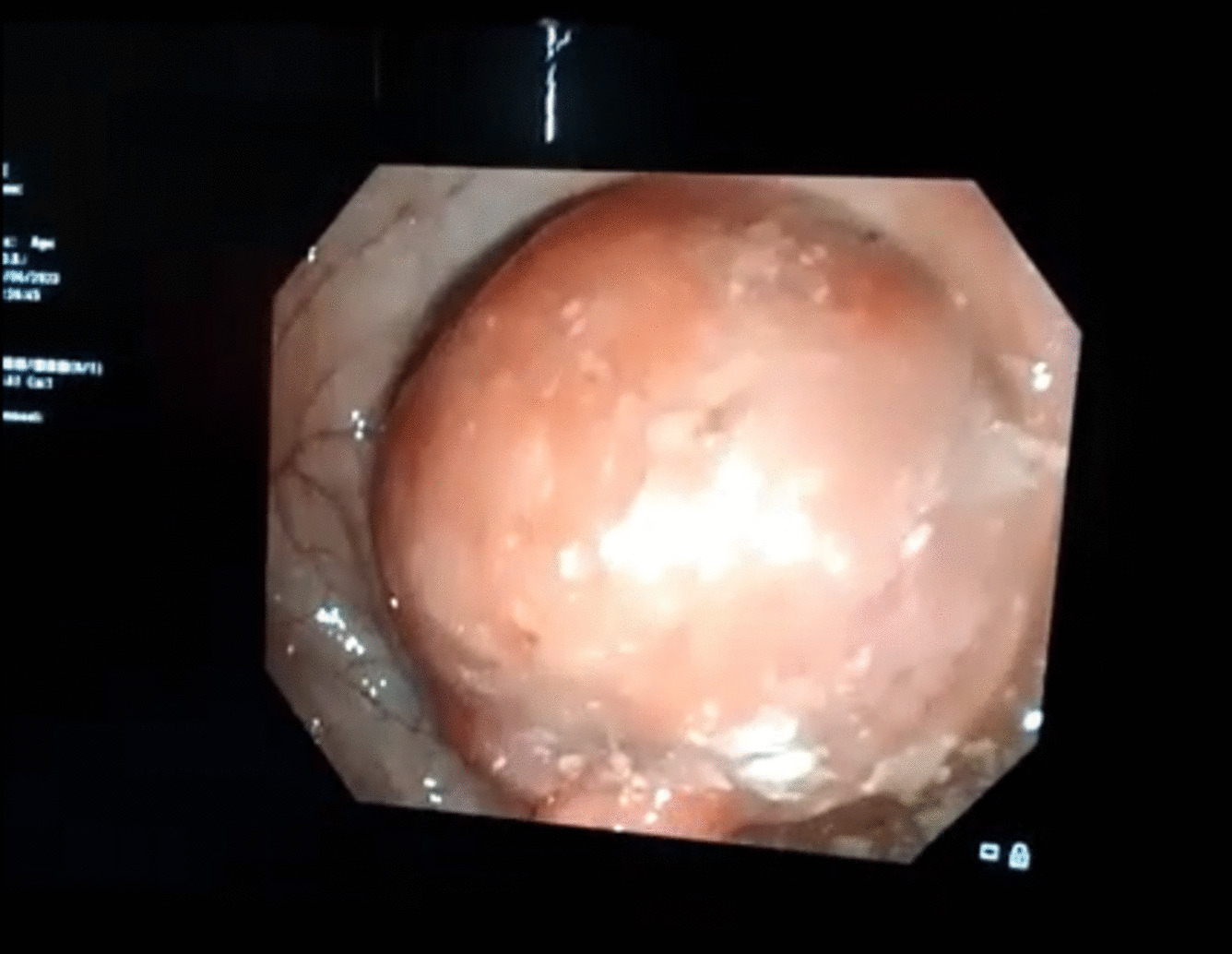
Colonoscopic picture of large polyp occupying the whole lumen at the level of splenic flexure

On colonoscopy (Fig. 3), polypectomy of small one and large one at sigmoid region was carried out and sent for histopathology, which revealed hamartomatous polyp with no signs of malignancy.

On endoscopy, the impression was: single large pedunculated polyp in the antrum, single sessile polyp in the D1–D2 junction, and small polyp in D4 region.

On the second session of MDT, it was decided to follow patient on the basis of PJS follow-up protocols and intervene only if the patient presented with any sign of obstruction or complications.

Only 1 week after discharge, she was again admitted with severe abdominal pain in the left flank and relative constipation. She was assessed by MDT, and it was decided to proceed with colonoscopic assessment. On colonoscopy, one giant polyp in hepatic flexure was resected, but on the same day the patient’s abdominal complaints became more severe. Erect chest X-ray showed air under diaphragm, and on examination patient had generalized peritonitis. On the basis of assessment, exploratory laparotomy was performed. Intraoperatively there was perforation in the hepatic flexure with no fecal soiling. After thorough assessment, extended right hemicolectomy with double barrel stoma was performed along with removal of giant broad-based polyp in duodenum, which was excised, and primary closure of duodenum was performed. Postoperatively, the patient was doing well, discharged on third postoperative day, and called for follow-up visits.

## Discussion

Hamartomatous polyps in the gastrointestinal tract may result in bleeding and small bowel intussusception, which may necessitate immediate surgical intervention [2–10].

In PJS, small intestinal polyps are the primary cause of intussusceptions. Most cases that have been documented in the literature are ileal or jejunal. Due to its permanent retroperitoneal position, the duodenum is a rare site for intussusception [11].

In our case there were intussusceptions in the transverse colon as well as the small bowel. A single large polyp in the pylorus and duodenum was also present in our patient, but there were no signs of intussusception.

The clinical diagnosis of PJS is established with one of the following: [12]

- Two or more histologically confirmed PJS-type hamartomatous polyps.
- Any number of PJS-type polyps detected in an individual who has a family history of PJS in at least one close relative.
- Characteristic mucocutaneous pigmentation in an individual who has a family history of PJS in at least one close relative.
- Any number of PJS-type polyps in an individual who also has characteristic mucocutaneous pigmentation.

In our case, all the criteria were fulfilled. Patient had hamartomatous polyp with positive family history of polyps of same features and mucocutaneous pigmentation on the lower lip, also a typical feature of PJS. Her niece was also positive for mucocutaneous pigmentation on the lower lip.

The following most frequent cancers associated with PJS are those of the breast, small bowel, gastric, and pancreatic organs [4–6, 10, 11]. In contrast to the general population, whose lifetime risks for any disease range from 37% to 93%, the lifetime cumulative cancer risk for this population ranges from 9.9% to 18% [13].

Polyp removal serves as the standard therapy to prevent complications. In fact, timely polypectomy can eliminate the necessity for repeated urgent surgeries and extensive resections of the small bowel, thereby reducing the risk of developing short bowel syndrome [14].

In our case, the patient also underwent polypectomies multiple times to avoid extensive surgery for resection of bowel.

The likelihood of complications in Peutz–Jeghers syndrome is intended to be decreased by periodic surveillance and removal of larger polyps. Thus, as recommended by the clinical guidelines of the American College of Gastroenterology, patients should have an annual complete blood count in addition to an annual physical examination that includes an examination of the testes, abdomen, and pelvis. Lifelong cancer surveillance is recommended. At the age of 8–10 years, surveillance for stomach and small bowel polyposis should start, and it should continue every 2–3 years [15].

Our MDT panel also decided to follow standard surveillance protocol for patient accordingly.

Genetic evaluation of a patient with possible PJS should include testing for *STK11* mutations [16]. In our case it was not done because of non-availability of testing facility.

The only limitation of this study is that we were unable to get *STK11* genetic analysis of the patient due to lack of facility of testing.

## Conclusion

Overall, this case report emphasizes the clinical features, diagnostic criteria, management strategies, and surveillance protocols for PJS. Further research and genetic testing are warranted to enhance the understanding and management of this rare inherited autosomal disorder.

## Data Availability

On request.
